# Evaluating Spatially Resolved Influence of Soil and Tree Water Status on Quality of European Plum Grown in Semi-humid Climate

**DOI:** 10.3389/fpls.2017.01053

**Published:** 2017-06-20

**Authors:** Jana Käthner, Alon Ben-Gal, Robin Gebbers, Aviva Peeters, Werner B. Herppich, Manuela Zude-Sasse

**Affiliations:** ^1^Horticultural Engineering, Leibniz Institute for Agricultural Engineering and Bioeconomy (ATB)Potsdam, Germany; ^2^Institute of Soil, Water, and Environmental Sciences, Agricultural Research Organization, Gilat Research CenterGilat, Israel

**Keywords:** fruit quality, precision horticulture, plum, spatial variability, tree water status

## Abstract

In orchards, the variations of fruit quality and its determinants are crucial for resource effective measures. In the present study, a drip-irrigated plum production (*Prunus domestica* L. “Tophit plus”/Wavit) located in a semi-humid climate was studied. Analysis of the apparent electrical conductivity (ECa) of soil showed spatial patterns of sand lenses in the orchard. Water status of sample trees was measured instantaneously by means of leaf water potential, Ψ_leaf_ [MPa], and for all trees by thermal imaging of canopies and calculation of the crop water stress index (CWSI). Methods for determining CWSI were evaluated. A CWSI approach calculating canopy and reference temperatures from the histogram of pixels from each image itself was found to suit the experimental conditions. Soil ECa showed no correlation with specific leaf area ratio and cumulative water use efficiency (WUEc) derived from the crop load. The fruit quality, however, was influenced by physiological drought stress in trees with high crop load and, resulting (too) high WUEc, when fruit driven water demand was not met. As indicated by analysis of variance, neither ECa nor the instantaneous CWSI could be used as predictors of fruit quality, while the interaction of CWSI and WUEc did succeed in indicating significant differences. Consequently, both WUEc and CWSI should be integrated in irrigation scheduling for positive impact on fruit quality.

## Introduction

Following the concept of precision agriculture, correlation of spatial variation of soil and yield data has been analyzed in field crops, vegetable production, vineyards, and orchards. Spatial patterns of fruit yield are typically explained in one of two approaches. The first analyzes the spatial correlation between soil properties influencing the water supply as one main growth factor and yield as the target variable. This is consistent with findings in precision viticulture, where soil maps have provided a basis for delineating management zones (Williams and Araujo, 2002). The second approach is more driven by the endogenous growth factors of the plant. It uses the correlation of plant data such as canopy volume representing the growth capacity, tree water status, and fruit quality at harvest (Zaman and Schumann, 2006). This latter approach may be more appropriate for orchards where fruit quality is crucial for marketing. However, the analysis of spatially-resolved soil and plant data and its influence on fruit quality has rarely been studied.

The most common method for soil mapping is to analyze the apparent electrical conductivity (ECa) of the soil (Bramley and Hamilton, 2004). Soil ECa measurements can be performed at field capacity to gain information regarding texture of the soil, while measurements in dry periods may better indicate soil water distribution. Mapping of electrical properties in orchard soils appears not without its challenges as commercial rolling systems often fail to measure close to the trees. Manually performed readings, most often with equidistant Wenner array, have been used with more success in covering the entire orchard soil (Halvorson and Rhoades, 1976; Gebbers et al., 2009). Experimental-scale ECa mapping, concomitantly performed with fruit yield analyses, confirmed a correlation between soil patterns and yield in various fruit crops including apples (Türker et al., 2011; Aggelopoulou et al., 2013), olives (Fountas et al., 2011; Agam et al., 2014), and citrus (Zaman and Schumann, 2006; Peeters et al., 2015). However, while patterns of soil properties are generally stable over time (Mann et al., 2011), spatial patterns of variables measured on trees are more likely to vary (Aggelopoulou et al., 2013). Furthermore, in orchards, soil water status is frequently influenced by irrigation causing intentionally reduced impact of a-priori patterns of soil properties on vegetative and generative plant growth. As a result, the effect of soil patterns on the quality of fruit might be reduced.

Using a physiological approach, the spatial variability of yield and quality have been found to be highly correlated with the canopy volume in citrus production (Zaman and Schumann, 2006; Zude et al., 2008). From a physiological point of view, it may be assumed that canopy volume, yield, and fruit quality are influenced by the exogenous water supply and the endogenous crop load (Palmer, 1992; Naor et al., 2001, 2006; Bustan et al., 2016). Strong interaction between water status of soil and trees has been pointed out in arid and semi-arid conditions (Naor et al., 2006; Ben-Gal et al., 2009; Gómez-del-Campo, 2013; Bustan et al., 2016), but also more ambiguous effects of crop load on tree water status have been reported for crops including peach, apple, and olive (Berman and DeJong, 1996; Bellvert et al., 2016; Bustan et al., 2016). The ultimate objective of orchard management of course would be to optimize not only the fruit quality, but also the cumulative water use efficiency (WUEc) in terms of yield per liter of totally applied irrigation and precipitation water (Viets, 1962).

The measurement of both, soil water status and plant water status, is challenged by the fact that any individual proximal sensor represents only a small volume of interest; a tree or part of a tree or a small volume of soil. Consequently, measuring the spatial distribution of water status in fruit trees has been approached by means of remote sensing, often via thermal imaging. Thermal images of canopies provide a measure of instantaneous tree water status interpreted by means of the crop water stress index (CWSI; Jones, 1992). The CWSI is a surface-temperature based index between 1 and 0, with 1 representing the temperature of non-transpiring dry leaves and 0 equivalent to that of fully transpiring wet leaves (Jackson et al., 1981; Sammis et al., 1988; Maes and Steppe, 2012). While application of thermal imaging is easily applied in the laboratory, the technique has also been developed for field studies, particularly in the semi-arid and arid sub-tropics (Jones, 1992; Cohen et al., 2005; Hellebrand et al., 2006). Thermal imaging of canopies has been applied by means of unmanned aerial systems (Berni et al., 2009; González-Dugo et al., 2013) and frequently tractor-mounted cameras providing either top or side views. The method has further been refined to measure CWSI and guide irrigation protocols in olives in Israel (Ben-Gal et al., 2009). In peach orchards located in a semi-arid environment, the CWSI was found to successfully differentiate between irrigation treatments (Bellvert et al., 2016). In differently irrigated apple trees under a hail net, CWSI values ranged between 0.08 and 0.55. Values >0.3 were considered as stressed trees under the given conditions (Nagy, 2015). The development and use of CWSI has focused on sub-tropical, arid, and semi-arid climates and has not yet been sufficiently studied under semi-humid conditions, where improving fruit quality, instead of providing for canopy transpiration, may be the most significant driver of irrigation water management. It is questionable if instantaneous methods for measuring water status, such as the thermal based CWSI, can support optimization of fruit quality on one hand and WUEc on the other side.

Consequently, this study aimed (i) to select a feasible method for utilization of thermal imaging in a semi-humid climate, (ii) to spatially characterize the soil ECa and instantaneous water status of fruit trees in an orchard, and (iii) to analyze the interaction of tree water status and quality of fruit.

## Materials and methods

### Site description and plant material

The experiment was carried out in a 0.37 ha commercial *Prunus domestica* L. (plum) orchard located in the “Werder fruit production” area in Brandenburg, Germany (52° 28′ 1.56″ N, 12° 57′ 28.8″ E). The soil is typical for fruit production in temperate climate of Europe and Asia formed by glacial and post-glacial deposits after the last ice age about 10,000 years ago with typically small scale variability. The cultivar was “Tophit plus” with “Jojo” serving as a pollinator. One hundred and four 7 year old “Tophit plus” trees, located every 4 m in 4 rows spaced 5 m apart, were considered. On average, trees were 2.10 m tall and insertion height of the first branch varied between 0.46 and 0.96 m above the soil. Mean soil texture was 45% sand, 29% silt, and 26% clay with a mean pH of 7.72. Plum trees were irrigated using a drip system with one line per row and two emitters every 0.5 m. The irrigation laterals and drippers were mounted 50 cm above the ground to facilitate mechanical weed control. Independent of precipitation, trees were irrigated twice a week for 1.5 h with flow rate of 0.96 L h^−1^.

### Meteorological readings

Global radiation, wind speed, air temperature, air pressure, precipitation, and relative humidity were measured at 24 min intervals by a weather station (UNIKLIMA vario, Toss, Germany) positioned 100 m from the experimental orchard. Canopy temperature and relative humidity (Modul DLTi, UP GmbH, Germany) were recorded in 18 trees every 5 min. Water vapor pressure deficit (VPD) of the air was calculated according to the Goff–Gratch-equation (Jones, 1992; von Willert et al., 1995) from hourly averages of air temperature, relative humidity, and air pressure.

### Soil properties

A resistivity meter (4-point light hp, LGM, Germany) was used to map the ECa of the soil at the experimental site on 16th August 2012 and 2nd August 2013. The four electrodes were arranged in a Wenner array with the tree trunk in the center to obtain ECa values representing 25 cm depth (Telford et al., 1990). Full details are given in Käthner and Zude-Sasse (2015). Soil water matric potential (pf-meter 80, ecoTech Umwelt-Messsysteme GmbH, Germany) was measured at 15, 35, and 45 cm depths. In addition, the gravimetrical soil water content (GWC) was ascertained by drying soil samples at 105°C for 48 h with *n* = 26 in 2012 and *n* = 6 in 2013.

### Leaf water status

Three mature leaves were randomly detached from the north-eastern side of each tree and rapidly transported to the laboratory. Here, projected surface area [cm^2^] was measured for each leaf with a portable area meter (CI-203, CID Bio-Science, Inc., USA). Leaf dry mass [g] was consequently obtained after oven drying at 65°C for 24 h and specific leaf area (SLA) was calculated as the ratio of leaf area and dry mass.

In the orchard, leaf water potential (Ψ_leaf_) was measured with a Scholander bomb (Plant Water Status Console 3000, Soilmoisture Equipment Corp., USA) on three shaded leaves from the lower part of the canopy on the east side of the tree. In 2012, 44 trees were analyzed predawn and midday over 4 days (19th June–27th June). In 2013, 67 trees were sampled over 5 days (19th July–2nd August). Following determination of Ψ_leaf_, the leaves were rapidly packed in plastic bags, transported to the laboratory, frozen at −30°C. After thawing, centrifuged tissue sap was analyzed for osmotic content (c_osmol_) with a water vapor osmometer (Vapro 5520, Wescor Inc., USA). The osmotic potential (Ψ_π_) of tissue sap was calculated according to the van't Hoff's equation (von Willert et al., 1995).

### Crop water stress index

Thermal images of the canopies were taken with an uncooled infrared thermal camera (ThermaCAM model SC 500, FLIR Systems, Inc., USA) with resolution of 320 × 240 pixel and spectral sensitivity range from 7.5 to 13.0 μm in the temperature range of −50 to 60°C on 15th August 2012 and 25th July 2013. The camera was mounted on a tractor with *z* = 3.3 m above ground and pointed to the top of the canopies. Images were acquired with an opening angle (β) of 45°resulting in the length (l) of the imaged area (Equation 1).

(1) $$\begin{array}{r} \text{l}=2\cdot \text{ z}\cdot \text{tan}\left(\frac{\beta }{2}\right) \end{array}$$

For extraction of temperature values, the raw thermal images were obtained in the FLIR systems' proprietary format and converted to text file format for the processing with MATLAB® (R2010B, MathWorks, USA). Crop water stress index (CWSI_J_) was calculated (Equation 2) according to Jones (1992) ranging from 0 to 1:

(2) $$\begin{array}{r} \text{CWS}{\text{I}}_{\text{J}}=\frac{\text{Tc}-\text{Twref}}{\text{Tdref}-\text{Twref}} \end{array}$$

where Tc is actual canopy temperature, Tw is temperature of a fully transpiring leaf with open stomata obtained from a wet paper leaf analog, and Td is temperature of a non-transpiring leaf. When using references Td_ref_ was obtained from a dry and Tw_ref_ from paper leaf analog (Jones, 2004). For this purpose, green paper leaves were cut to the formerly measured mean leaf area of 6 cm^2^, mounted on a 2 m stick, and manually placed in the center of the canopy in each tree.

In addition, CWSI was also calculated according to three alternative methods. Irmak et al. (2000) calculated the CWSI_I_ (Equation 3) setting non-transpiring leaf temperature at 5°C higher than air temperature (Td+5) and Tw as the minimum temperature found in the canopy.

(3) $$\begin{array}{r} \text{CWS}{\text{I}}_{\text{I}}=\frac{\text{Tc}-\text{Twmin}}{\text{Td}+5-\text{Twmin}} \end{array}$$

As described by work groups of Jones (1999) and Ben-Gal et al. (2009), Tw and Td was obtained analytically (Appendix in Supplementary Material) to calculate CWSI_JB_ (Equation 4).

(4) $$\begin{array}{r} \text{CWS}{\text{I}}_{\text{JB}}=\frac{\text{Tc}-\text{Twana}}{\text{Tdana}-\text{Twana}} \end{array}$$

CWSI_R_ was determined according to the work of Rud et al. (2015). Likely the most suitable for automated readings, this method calculates (Equation 5) the canopy temperature (Tc_histo_) and reference temperatures of dry (Td_histo_) and wet (Tw_histo_) leaves from the histogram of pixels from each image itself.

(5) $$\begin{array}{r} \text{CWS}{\text{I}}_{\text{R}}=\frac{\text{Tchisto}-\text{Twhisto}}{\text{Tdhisto}-\text{Twhisto}} \end{array}$$

In the CWSI_R_ approach, before processing histograms, extreme values above air temperature representing Fresnel reflection from the sun were removed from the further analysis. In the histogram of pixels, thresholds were determined for separating temperatures of soil, grass, and canopy. Dry reference, Td_histo_, was defined as the minimum temperature of soil visible as a peak with high values in the histogram. Wet reference, Tw_histo_, was taken as the minimum temperature of canopy. Since the canopy and grass partly coincided, pixels were spatially compared considering equal values as grass and varying values as canopy. This threshold was found with Wiener filter to enhance the contrast (Honig and Goldstein, 2002; Chen et al., 2006). After removing the soil and grass data, the Td_histo_ and mean canopy temperature (Tc_histo_) were extracted and averaged for each tree.

### Water use efficiency

On the day of CWSI measurement, a portable porometer (CIRAS-1, PP Systems, Hitchin, UK) was used to monitor the diurnal course (*n* = 3) of CO_2_ exchange and transpiration. The instantaneous water use efficiency (WUE_*i*_) was calculated as ratio of these parameters (von Willert et al., 1995) in μMol CO_2_ m^−2^ s^−1^/mMol H_2_O m^−2^ s^−1^.

The WUEc (Equation 6) of the production system represents the ratio of yield (y) and water volume supplied to the plants [g L^−1^],

(6) $$\begin{array}{r} \text{WUEc}=\text{y}/\left(\text{i}+\text{pp}\right) \end{array}$$

with i = irrigation water, pp = precipitation from the start of vegetation period until harvest time.

In 2012, the accounted period lasted from 17th April to 30th August during which 182 mm of irrigation and 273 mm rain with a total of 455 mm water were supplied. In 2013, irrigation water was given from 22nd April to 9th September accounting for 168 mm of irrigation water and 248 mm of rain was recorded summing up to 416 mm water supply.

### Fruit quality

Soluble solids content [%] of fruit was analyzed using a digital refractometer (DR 301-95, A. Krüss Optronic, Germany). Dry matter content of fruit [%] was calculated as the ratio of fruit dry mass and fruit fresh mass. Fruit flesh firmness [N cm^−2^] was analyzed as maximum force measured with a convex plunger at a velocity of 200 cm min^−1^ (TA-XT Plus Texture Analyzer, Stable Micro Systems, UK). Fruit size measured as height [mm], fresh mass [g], and yield as number of fruits per tree and fresh mass per tree, was measured at harvest. In 2012, the analysis of fruit quality was carried out on all fruit of every tree, while in 2013, 3 fruits per tree were analyzed.

### Data analysis

Statistical analyses were carried out using the statistical package for MATLAB® (R2014b, MathWorks, U.S.). Multi-way analysis of variance (ANOVA) was used for testing the effects of multiple factors on the plant variables. Therefore, the ECa data were grouped in 8 classes (Käthner and Zude-Sasse, 2015), while CWSI and WUEc were grouped according to the results of hotspot analysis.

Descriptive statistics of spatially resolved data was carried out using hotspot analysis according to Peeters et al. (2015), who used ArcGIS (ESRI, Redlands, CA, USA). In the present study, the algorithm was adapted for using the free spatial Matlab toolbox (Spatial Filtering, Max Planck Institute for Biochemistry, Germany). The method is based on the general (G) statistic for testing the effect of spatial autocorrelation (Getis and Ord, 1992) of the variables. Thereby a locally weighted mean around each observation is separately compared with the mean of the whole data (Anttila and Kairesalo, 2010). The outputs of the statistic are the z-score and the *p*-value, which indicate whether an observed pattern of clusters is statistically significant. Spatial clusters with statistically significant positive z-score are called hot spots, whereas the clusters with statistically significant negative z-score are called cold spots (Getis and Ord, 1992; Ferstl, 2007).

## Results

### Soil, meteorological conditions, and thermal imaging

**Figure 1:**
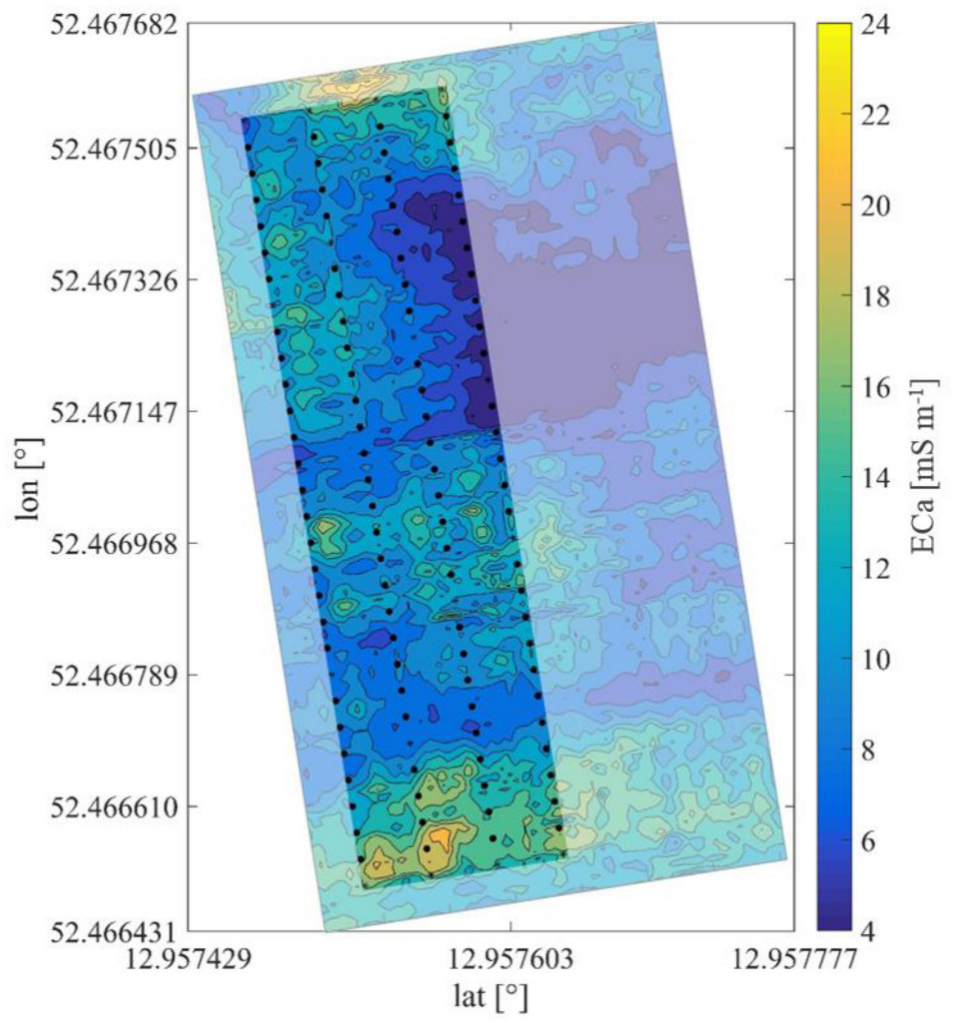
Plum orchard in north orientation with trees marked, showing apparent electrical conductivity of soil in false color.

The ECa of soil at 25 cm depth indicated small-scale variability (Figure 1). The values of soil ECa reached a maximum of 24 mS m^−1^ with a pattern of reduced values pointing to a sand lens visible in the center-eastern part of the experimental field (Figure 1) and neighboring area. Another sandy area was located in the south-west of the orchard. Values of soil ECa measured in 2013, increased compared to those obtained in 2012. This may be due to wetter soils, which was caused by the relatively high precipitation occurring in July and August 2013. This assumption is further supported by the close correlations found between the gravimetrical soil water content and ECa with *R* = 0.45 and *R* = 0.68 in 2012 and 2013, respectively (Table 1). In contrast, correlation coefficients of soil matric potential (pF) and soil ECa were only *R* = 0.15 and *R* = 0.44 in 2012 and 2013, respectively (Table 1). Repeated analyses showed similar pattern in different years with *R* = 0.88 considering 2011 and 2012 and *R* = 0.71 for years 2012 and 2013.

**Table 1:** Summary of soil properties measured in plum orchard.

**Variable**	***n***	**Mean**	**Minimum**	**Maximum**	**SD**	**Skewness**
**2012**						
ECa [mS m^−1^]	104	7.09	1.67	24.38	5.77	0.90
pF units [0;7]	19	1.63	0.04	2.10	0.44	−2.58
Water content [%]	26	7.61	4.43	9.63	1.37	−0.57
**2013**						
ECa [mS m^−1^]	180	32.43	8.89	83.89	13.69	0.75
pF-units [0;7]	19	1.70	0.01	3.30	0.98	−0.51
Water content [%]	6	18.58	9.14	31.13	4.07	0.25

**Figure 2:**
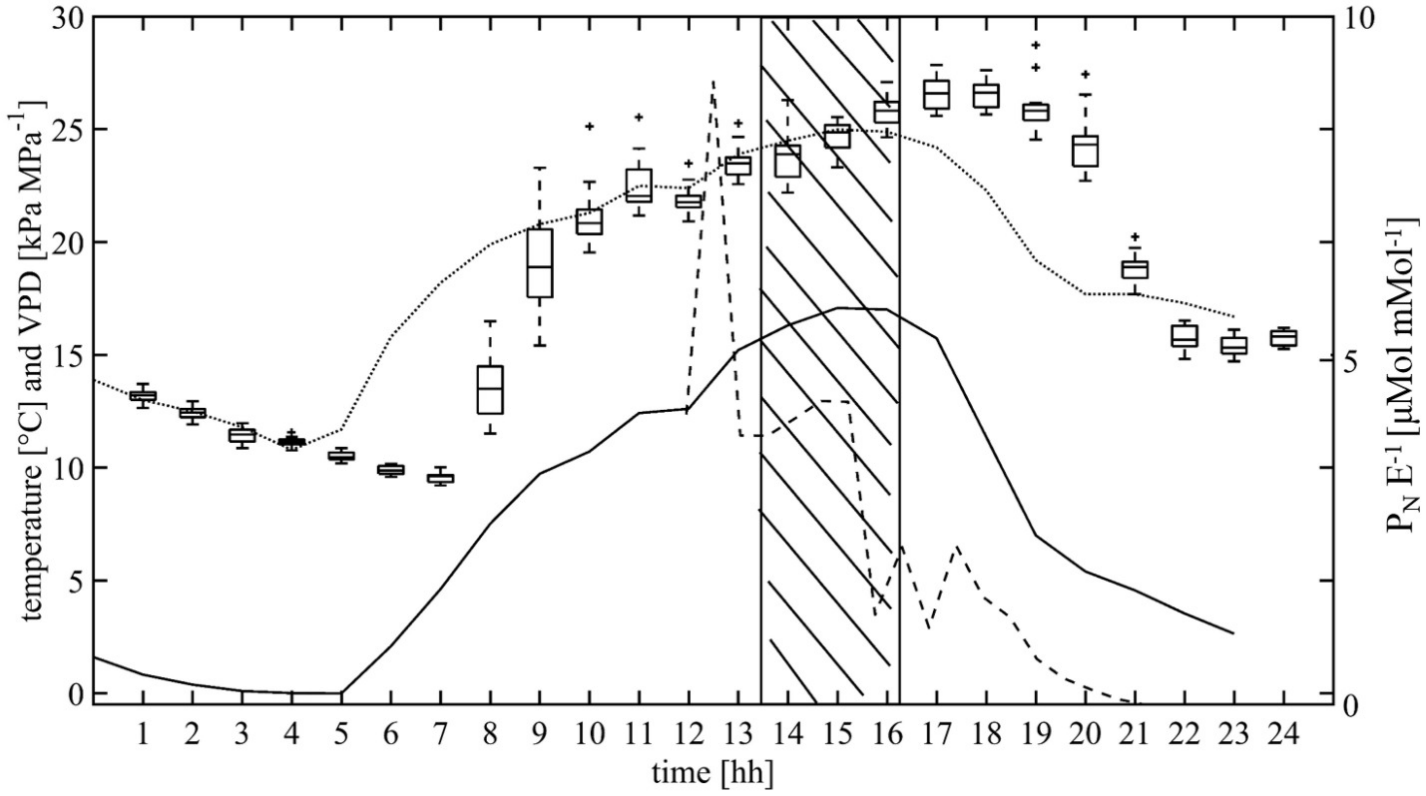
Air temperature (dotted line), water vapor pressure deficit (VPD; solid line) and instantaneous water use efficiency (WUE_i_ as P_N_ E^−1^, dashed line) measured in the orchard on 15th August 2012. In addition, the variation (*n* = 18) and diurnal course of tree canopy temperature is shown as boxplot. The dashed area indicates the period used for analyzing the CWSI.

In 2012, during the acquisition of thermal images on 15th August from 13:40 until 16:19 (Figure 2), the mean global radiation was 641.1 W m^−2^. August was the warmest month of the year with a mean maximum temperature of 25.6°C. The maximum air temperature on the day of measurement was 25.4°C. The diurnal increase of air temperature coincided with increasing VPD. The mean wind speed was 0.9 m s^−1^. In 2013, mean global radiation of 306.8 W m^−2^, maximum air temperature of 25.4°C, and wind speed of 1.2 m s^−1^ were measured.

Compared to the free air temperature recorded by the automatic weather station, maximum temperature measured within the tree canopy occurred with a 3 h-delay. Maximum instantaneous water use efficiency was calculated just before noon and then declined during the rest of the day. In general, it ranged from 4.42 to 1.28 μMol CO_2_ m^−2^ s^−1^/mMol H_2_O m^−2^ s^−1^ (Figure 2). Inside the canopy, the VPD increased midday reaching a maximum in the afternoon at 17:00.

The instantaneous Ψ_leaf_ at midday varied between −0.40 and −2.14 MPa and Ψ_π_ between −1.93 and −2.56 MPa. At predawn, Ψ_leaf_ varied between −0.12 and −1.48 MPa and Ψ_π_ between −1.50 and −2.46 MPa.

Thermal images were acquired on partially cloudy days and wet and dry leaf-references were moved with the camera for each tree record within the orchard. With our camera set-up, l = 2.734 m and thus one pixel corresponded to 8.543 mm in width. This resolution was, thus, high enough to differentiate leaves, and to select the pixels that represent the wet and dry leaf-references (Table 2). In 2012, Vaseline® covered leaves were additionally used as dry leaf-references; however, the fingerprints of the application procedure remained visible on thermal images thus producing artifacts (data not shown).

**Table 2:** Ranges of wet (Tw) and dry (Td) reference temperatures obtained according to work groups of Jones and Ben-Gal (Ben-Gal et al., 2009) using weather data (CWSI_JB_), Jones (Jones, 1992) using dry and wet paper leaves (CWSI_J_), and Rud (Rud et al., 2015) using both references from the histogram of image (CWSI_R_).

**Variable**	***n***		**CWSI_JB_**	**CWSI_J_**	**CWSI_R_**
Tw			12.07–15.03	19.98	16.10–20.80
Td			16.95–17.06	24.93	21.00–24.00
ψ_leaf_	11	R	−0.65	−0.12	−0.52
		F	4800^***^	3671^***^	3671^***^
ψ_π_	11	R	−0.57	0.33	−0.11
		F	872^***^	911^***^	911^***^

Correlation coefficients (R) and F-values, asterisks (^***^) denoting significance at p < 0.001 considering leaf water potential (ψ_leaf_), osmotic potential (ψ_π_), and crop water stress indexes are given of the same measuring day.

The reference temperatures calculated with the analytical method (Ben-Gal et al., 2009) were always lower (Tw_ana_ 12–15 and Td_ana_ 17) compared to those measured on paper references or obtained from the histogram of images (Tw_histo_ 16–21°C and Td_histo_ 21–25°C). Furthermore, the correlations between leaf water potential (ψ_leaf_) or osmotic potential (ψ_π_) and the different crop water stress indexes were analyzed using data that were all obtained on the same day. Of all tested approaches, correlation coefficients for both ψ_leaf_ and ψ_π_ were highest for CWSI_JB_, i.e., when the dry and wet temperatures were calculated analytically. In contrast, correlation between CWSI_J_ and ψ_leaf_ was low, showing enhanced variability caused by the appearance of clouds (Table 2). The use of air temperature plus 5° as Td and minimum temperature in the image as Tw for calculating CWSI_I_ (Irmak et al., 2000) resulted in a bias with overestimated values and also tremendously high variability due to clouds and, therefore, data were not used further. The CWSI_R_ ranged from 0.15 to 0.88, while the CWSI_J_ and CWSI_JB_ ranged from 0.03 to 0.78 and from 0.47 to 0.51, respectively. For CWSI_JB_ correlation with ψ_leaf_ was high, while the automated analysis of CWSI_R_ resulted in slightly reduced, but significant (*p* < 0.001) correlation coefficient of *R* = 0.52 (Table 2). However, the latter approach provided the advantage of feasible analysis of Td and Tw based on the individual images taken in the varying environment. Consequently, all further analyses were based on CWSI_R_.

### Hotspot analyses

**Figure 3:**
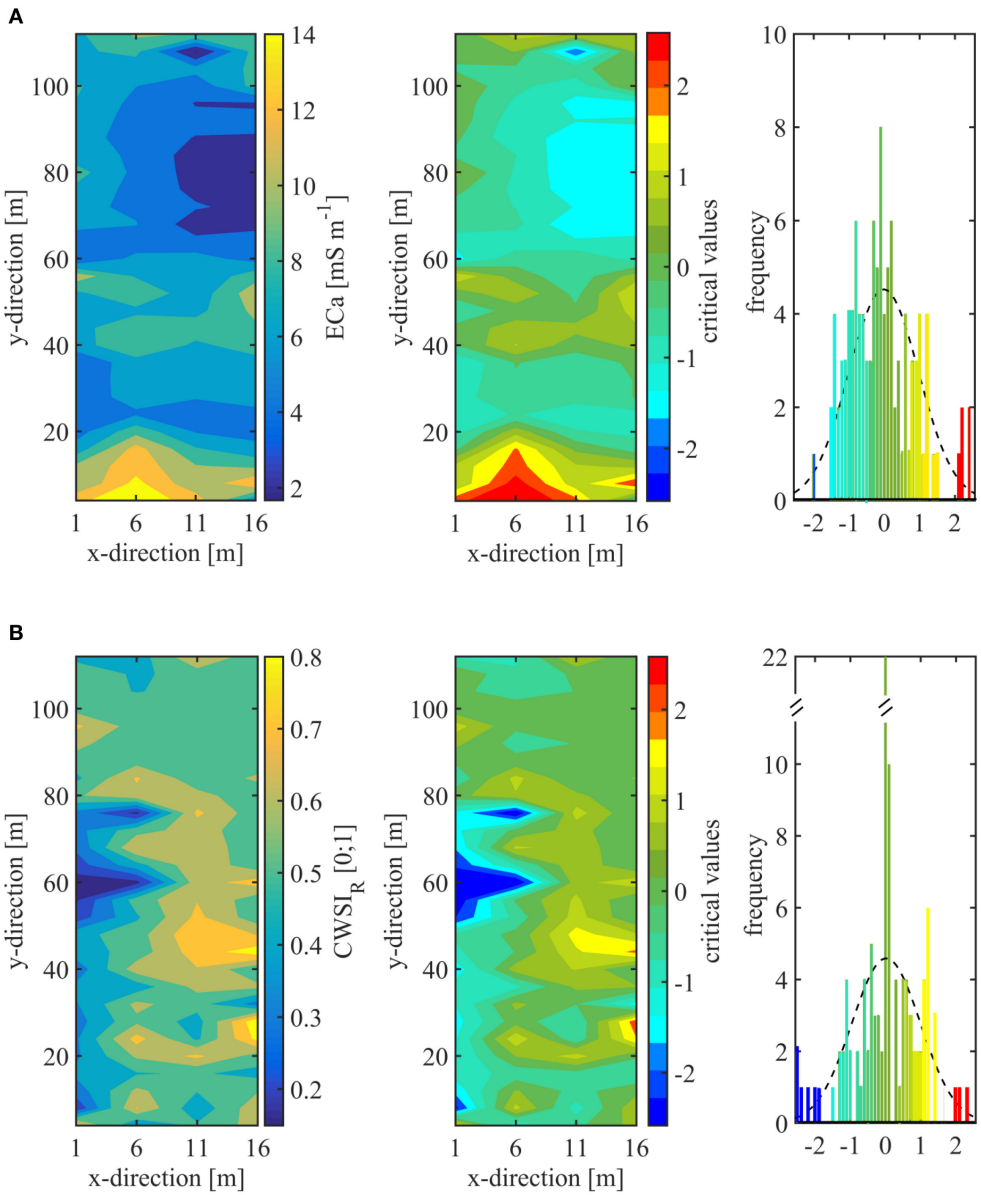
False color maps providing the spatial distribution of **(A)** soil apparent electrical conductivity (ECa) and **(B)** instantaneous tree water status measured as crop water stress index (CWSI_R_) in the experimental plum orchard. Given are raw data (left), critical values by hotspot analysis (middle), and histograms of critical values (right).

Hotspot analysis of ECa revealed one cold spot representing extreme low conductivity and 5 hot spots showing soil of high conductivity. Around the cold spot with critical *z*-value of < −1.65 (90% confidence level) a sand lens with an extension of ~20 × 25 m was found (Figure 1), while at the hot spots with critical value >1.65 (90% confidence level) water logging was observed after heavy rain fall indicating soil with lower particle size (Figure 3). The soil ECa was correlated with the number of leaves per tree. Consistently, spatial variability of canopy VPD within the orchard was found in the x-direction, which pointed to an influence of geographical position in the orchard (Rx = 0.31, Ry = 0.03, Rz = 0.20). This is the same direction as found for extreme values of soil ECa.

The CWSI_R_ ranged from 0.15 to 0.88. The hotspot analysis of CWSI_R_ revealed 5 cold spots occurring at *z*-values < −1.65 representing trees with no water shortage. The 3 hot spots appeared at critical value >1.65 referring to high CWSI_R_. Here, the hot spots refer to unfavorable conditions with enhanced water deficit. The hot spots appeared on the east side of the orchard, within and adjacent to the position of the central sand lens (Figure 3). The cold spots were found in the western positions of the orchard.

The comparison of spots considering soil ECa and CWSI_R_ pointed to no correlation. Also, no correlation was found between canopy size dimension and CWSI_R_ considering the canopy length parallel to the row (*R* = 0.010), canopy width perpendicular to the row (R = 0.015), and volume calculated from length, width, and distance between first branch and last shoot (*R* = 0.001).

### Tree water status and fruit quality

In 2012, the average leaf number per tree was 2,362. The SLA ranged from 32.00 to 59.76 cm^2^ g^−1^ and showed no correlation with soil ECa. The fruit size was correlated with soil ECa at *R* = 0.223 considering the hot and cold spots. However, other fruit quality variables did not correlate with soil properties.

No correlation between leaf water potential and fruit quality was found in the few trees measured. CWSI_R_ was correlated with SLA, but no significant difference was found for the number of leaves or fruit quality (Table 3). WUEc obviously depends primarily on the degree of crop load, because the water supply was kept uniform in the orchard. Mean WUEc was 2.362 g L^−1^ in 2012 and 2.521 g L^−1^ in 2013. In 2012, WUEc seemed only slightly, if at all, affected by soil ECa (*R* = 0.133), while in 2013, the correlation increased (*R* = 0.274).

**Table 3:** Mean values and *p*-level of plant variables grouped according to low (cold spot), random, and high (hot spot) crop water stress index (CWSI_R_) and cumulative water use efficiency (WUEc) considering mean values of all fruits and leaves of each tree.

**Variable**	**CWSI_R_ cold spot**	**CWSI_R_ random**	**CWSI_R_ hot spot**	***p***	**WUEc cold spot**	**WUEc random**	**WUEc hot spot**	***p***
# Leaves per tree	1973	2341	2734	0.589	2181	2399	2266	0.568
Specific leaf area [cm^2^ g^−1^]	na	47.07	49.27	0.023	49.05	49.29	48.48	0.730
Fruit size [mm]	58.22	55.04	54.67	0.670	59.82	54.79	52.34	<0.001
Firmness [N cm^−2^]	3.59	2.70	2.80	0.635	3.29	2.75	2.23	0.109
Dry matter [%]	33.97	32.37	32.08	0.393	32.30	32.27	32.96	0.031

**Figure 4:**
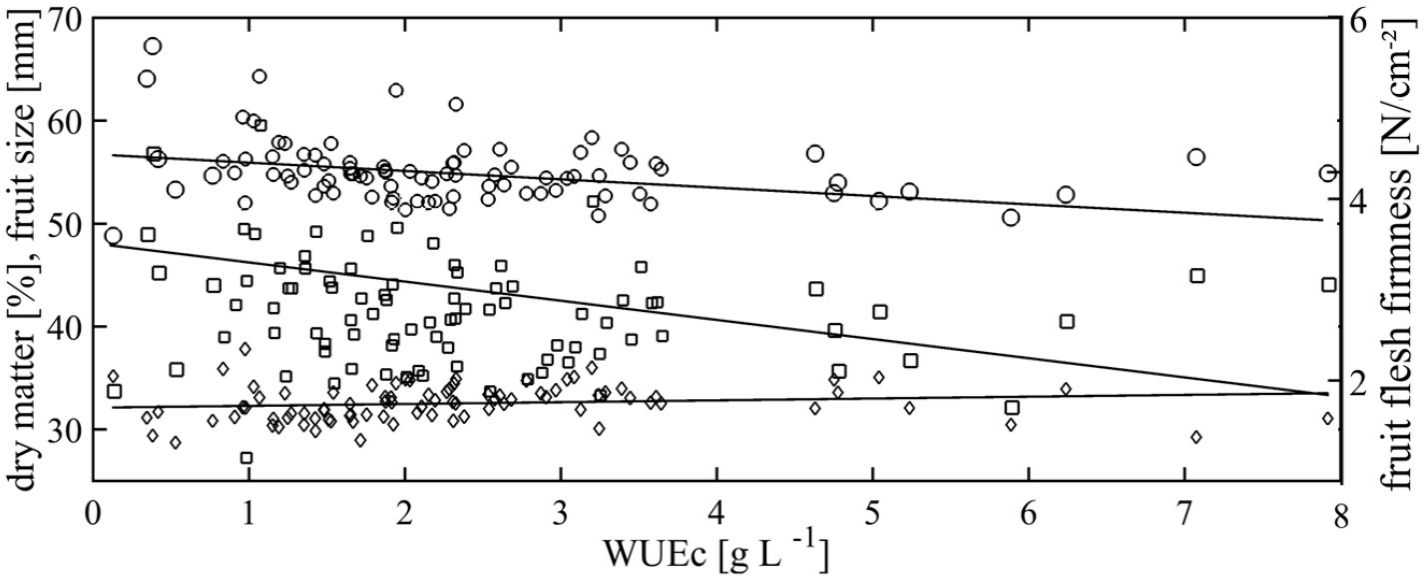
Regression analyses of data (means per tree; *n* = 88) of fruit dry matter (diamonds, y = 5.903x_1_ + 34.67), fruit size (circles, y = −80.17x_2_ + 24.29), fruit flesh firmness (squares, y = −0.129x_3_ + 3.017), and cumulative water use efficiency (WUEc). Increased symbol size represents cold and hot spots.

The WUEc showed a correlation of *R* = −0.367, *R* = 0.183, and *R* = −0.270 with the fruit size, dry matter, and fruit flesh firmness, respectively, in 2012 (Table 3). Particularly, larger fruit size was correlated with low WUEc, and consequently with decreased crop load (Figure 4). Correlation between the above parameters seemed to be stronger in 2013. However, the reduce sample size in 2013 hampered the statistical comparison of the influence of slight drought stress on fruit quality in the different years.

WUEc showed no correlation with CWSI_R_ with *R* = 0.071 and *R* = 0.093 in 2012 and 2013, respectively. However, fruit quality was strongly affected considering the interaction of both variables (Table 4). Grouping according to WUEc and the instantaneous values of CWSI_R_ resulted in highly significant differences for fruit size and dry matter.

**Table 4:** Interaction of cumulative water use efficiency (WUEc) × crop water stress index (CWSI_R_) and its effect on fruit quality analyzed by 2 factorial ANOVA considering all data and data excluding hot and cold spots.

**Variable of fruit quality**	**WUEc × CWSI_R_ of all data**		**WUEc × CWSI_R_ without spots**	
	***F***	***p***	***F***	***p***
Fruit size [mm]	1.94	<0.0001	1.89	<0.0001
Dry matter [%]	1.91	<0.0001	1.82	<0.0003
Firmness [N cm^−2^]	1.16	0.2178	0.5	0.9977

## Discussion

### Spatial patterns in the orchard

Shortly before harvest, the spatial patterns of soil ECa appeared closely related to soil water content with decreased ECa values at the positions of a sand lens found in the experimental orchard. This finding is consistent with earlier investigations carried out in areas with arid conditions (McCutcheon et al., 2006). The low correlation between soil matric potential and soil apparent electrical conductivity could be expected because previous chemical analyses of soils (Käthner and Zude-Sasse, 2015) at 10 spots of the same experimental site indicated only marginal <5% variations of phosphorus and potassium content, salinity, and pH. Increased, but still <10% variation was found for magnesium, calcium, sodium, and chloride contents. Nevertheless, the analyses of the variations of soil ECa during fruit development may provide data and information for the evaluation of spatial distribution pattern of water, which could potentially affect the quality of the mature fruit.

The Ψ_leaf_ measured predawn showed high variability and minimum value of −1.48 MPa indicating at least slight drought stress in some trees. Based on the measurements of weather and tree canopy microclimate, stable environmental conditions (Bellvert et al., 2014) during thermal imaging between 13:40 and 16:19 can be assumed for both years. Only the variation of radiation due to changing cloud cover could have slightly impaired thermal imaging due to the different dynamics of surface and air (ambient) temperatures (Agam et al., 2013). On the other hand, the analysis of the instantaneous WUEi, performed at the same time, revealed diurnal changes in a value range reported in other investigations on plum trees under similar conditions (Flores et al., 1985). Consequently, consistent sets of thermal readings may have been obtained on each measurement day.

The influence of clouds indeed appeared as a perturbing factor in the present study, especially when using dry and wet paper as references for obtaining Tw and Td. The use of air temperature plus 5 K for setting Td with low difference of Td and Tw resulted in high bias of CWSI_I_. The analytical analysis of Td and Tw, as well as the automated approach resulted in significant correlation of CWSI and Ψ_leaf_. Calculating Td and Tw analytically (Jones, 1999; Ben-Gal et al., 2009) had the disadvantage that weather data were needed. However, this method provided some insurance against artifacts. In the approach of intrinsic analysis of thermal images to calculate CWSI_R_ (Rud et al., 2015), references are directly obtained from the images, which is presumably the most feasible approach for an application of thermal imaging in a real world orchard avoiding the need for additional measurements. The approach appeared to be appropriate for the semi-humid summer rain region with cloudy conditions of the current study. The correlation coefficient of *R* = −0.52 considering Ψ_leaf_ and CWSI_R_ was at least encouraging to estimate the water stress of the plum trees.

Hotspot analysis (Getis and Ord, 1992) was applied to identify geographically located trees that differ from the mean. The spots found in the ECa data set point to significantly different clusters of trees appearing in the orchard. This small scale variability of soil ECa is typical for postglacial deposits which are common sources of soils in fruit production regions in temperate areas of Europe and Asia.

The CWSI_R_ varied between 0.15 and 0.88 presumably indicating a range of unstressed to stressed trees in the orchard. As for the ECa patterns, the appearance of significant clusters considering instantaneous CWSI_R_ points to a possible impact of tree water status on plant growth. However, we can certainly make no a-priori assumption on stable CWSI patterns, since crop load, stage of fruit development, and vegetative growth are all expected to influence water demand. This said, neither ECa nor crop load in the current study showed a correlation with CWSI_R_.

### Potential of irrigation adjustment for improving fruit quality

Bellvert and co-authors identified an influence of the fruit development stage on the correlation coefficient of leaf water potential and CWSI in peach and nectarine (Bellvert et al., 2016) with increased correlation shortly before harvest, which is developmental stage 3. In olive fruits, less severe but equally directed correlation was found (Martin-Vertedor et al., 2011). In the current study, CWSI_R_ was similarly measured in stage 3 of plum fruit development corresponding to the second peak of fruit growth rate with high water demands.

In plum production, fruit size is of highest economic importance. In the present study, no effect of instantaneous tree water status as indicated by CWSI_R_ on fruit size was found. However, at high crop load, fruit size was reduced and water required to produce high quality (large enough) fruits may have been deficient. While the instantaneous canopy transpiration based CWSI_R_ alone did not indicate this level of potential water deficit, cumulative data of WUEc was correlated with fruit quality.

The reducing effect of crop load on Ψ_leaf_ or stem water potential has been pointed out previously (Naor et al., 2001; Marsal et al., 2010), particularly under very high crop load (Sadras and Trentacoste, 2011). An impact on the fruit size is consequent. WUEc, by definition, was dependent of crop load, since, as said, the water supply was uniform in the orchard. However, the variability of soil ECa might point to differences in effective water supply, which would be worthwhile to consider in future studies for calculating the effective WUEc.

Considering the spatial variability measured in the present study, the factor combination of the cumulative WUEc and instantaneous CWSI_R_ resulted in highly significant interaction with fruit quality. The effects of WUEc and CWSI outweighed the effect of soil ECa on the fruit quality. However, these findings certainly need additional experimentation and confirmation before development as a practical management tool.

## Conclusions

Spatially resolved soil analysis is commonly applied in precision horticultural applications. In the present study, analysis of histograms of thermal images in a plum orchard located in a temperate climate characterized by cloud cover and semi-humid conditions was additionally confirmed as a feasible method for spatial quantification of water status.

Different spatial clusters of apparent electrical conductivity of soil and instantaneous CWSI were found, but none was correlated with fruit quality in the evenly irrigated orchard. While the WUEc showed an effect on fruit size, only combined analysis of instantaneous water status and WUEc yielded a close correlation with various fruit quality parameters. In practice, i.e., in model-based regulated deficit irrigation of orchards with frequently present small scale variability of soil and varying crop load, the coupled CWSI and WUEc, together with the stage of fruit development, is expected to be an effective driver.

## Author contributions

AB as an expert in irrigation of fruit trees in arid and semi-arid conditions contributed on the methodology of thermal imaging, including experimental set-up and CWSI analysis. He also added to the structuring and wording of the manuscript. AP as an expert in geospatial information systems introduced and supported the spatial descriptive statistical analysis. JK carried out the experiments and all statistical data analyses. She prepared the figures and tables and made a recent literature search and proposed the text. MZ as a horticulturist provided the objectives of the experiments, supervised the methodology, added the red line in the manuscript and supported the writing. RG as an expert in soil science supported the measurements of apparent soil electrical conductivity and data analysis. WH as a plant physiologist with focus on produce quality and particularly plant water status supported the analysis of tree water status.

### Conflict of interest statement

The authors declare that the research was conducted in the absence of any commercial or financial relationships that could be construed as a potential conflict of interest.

## Abbreviations

ρ: density of dry air [kg m^−3^]
t: time course, diurnal variation [h]
Ψ_leaf_: leaf water potential [MPa]
Ψ_π_: osmotic potential [MPa]
u: wind speed [m s^−1^]
T: temperature [°C]
T_K_: absolute temperature [K]
T_w_: temperature of wet reference [°C]
T_d_: temperature of dry reference [°C]
T_c_: current canopy temperature [°C]
T_air_: air temperature [°C]
CWSI: crop water stress index [0; 1]
ECa: soil apparent electrical conductivity [mS m^−1^]
VPD: water vapor pressure deficit [kPa]
WUE_i_: instantaneous water use efficiency [μMol mMol^−1^]
WUE_c_: cumulative water use efficiency [g/].
