# Supplementary material for: Evaluating Spatially Resolved Influence of Soil and Tree Water Status on Quality of European Plum Grown in Semi-humid Climate
Source: Front Plant Sci. 2017 Jun 20;8:1053. doi: 10.3389/fpls.2017.01053 (PMC5476784; doi:10.3389/fpls.2017.01053)
Supplement: Supplementary file 1 [file DataSheet1.docx]

**Appendix – Calculation of analytical analysis of wet and dry reference temperature**

$\mathbf{Twana=}\mathbf{T}_{\mathbf{air}}\mathbf{+}\frac{\mathbf{R}_{\mathbf{n}_{\mathbf{i}}}\mathbf{r}_{\mathbf{HR}}}{\boldsymbol{\rho}\mathbf{C}_{\mathbf{p}}}$ (1)

$\mathbf{Tdana=}\mathbf{T}_{\mathbf{air}}\mathbf{+}\frac{\mathbf{r}_{\mathbf{HR}}\mathbf{r}_{\mathbf{v}}\boldsymbol{\gamma}}{\boldsymbol{\rho}\mathbf{C}_{\mathbf{p}}\left( \mathbf{s}\mathbf{r}_{\mathbf{HR}}\mathbf{+}\mathbf{r}_{\mathbf{v}}\boldsymbol{\gamma} \right)}\mathbf{R}_{\mathbf{n}_{\mathbf{i}}}\mathbf{-}\frac{\mathbf{r}_{\mathbf{HR}}}{\mathbf{s}\mathbf{r}_{\mathbf{HR}}\mathbf{+}\mathbf{r}_{\mathbf{v}}\boldsymbol{\gamma}}\mathbf{VPD}$ (2)

Where ρ is the density of dry air [kg m^-3^], C_p_ the specific heat of dry air at constant pressure [J kg^-1^ K^-1^], γ the thermodynamic psychrometer constant (≈0.066 kPa K^-1^), r_v_ the aerodynamic resistance [s m^-1^] calculated by latent heat transport (r_H_/1.08), T_air_ the air temperature [K], the VPD [kPa], and s representing the slope of the curve relating saturation vapor pressure to temperature. The aerodynamic resistance to sensible heat transport r_H_ [m s^-1^] and the resistance for radiative heat loss r_R_ [s m^-1^] are compiled into the resistance to sensible heat transport r_HR_ [s m^-1^].

$\mathbf{r}_{\mathbf{H}}\boldsymbol{=100\surd}\frac{\mathbf{d}}{\mathbf{u}}$ (3)

$\mathbf{r}_{\mathbf{R}}\mathbf{=}\frac{\boldsymbol{\rho}\mathbf{C}_{\mathbf{p}}}{\boldsymbol{4\varepsilon\sigma}\mathbf{T}_{\mathbf{air}}^{\mathbf{3}}}$ (4)

Where d is the characteristic length [m] of the leaf in the direction of the prevailing wind, u represents the wind speed [m s^-1^], ε the emittance of the canopy with 0.99 (Jones, 2014) and σ refers to the Stefan-Boltzmann constant (5.67 * 10^-8^ W m^-2^ K^-4^).

$\mathbf{r}_{\mathbf{HR}}\mathbf{=}\frac{\mathbf{1}}{\frac{\mathbf{1}}{\mathbf{r}_{\mathbf{H}}}\mathbf{+}\frac{\mathbf{1}}{\mathbf{r}_{\mathbf{R}}}}$ (5)

The net energy at the canopy R_n_ [W m^-2^] is the sum of the incoming short-wave radiation R_SW_ [W m^-2^] and the incoming and outgoing long-wave radiations (Ben-Gal et al., 2009):

$\mathbf{R}_{\mathbf{n}}\mathbf{=}\mathbf{R}_{\mathbf{sw}}\left( \boldsymbol{1-\alpha} \right)\mathbf{+1.24(}\frac{\mathbf{10}\mathbf{e}_{\mathbf{air}}}{\mathbf{T}_{\mathbf{air}}}\mathbf{)}^{\frac{\mathbf{1}}{\mathbf{7}}}\boldsymbol{\sigma}\mathbf{T}_{\mathbf{air}}^{\mathbf{4}}\mathbf{-}\boldsymbol{\varepsilon}_{\mathbf{c}}\boldsymbol{\sigma}\mathbf{T}_{\mathbf{c}}^{\mathbf{4}}$ (6)

Where R_SW_ is the incoming short-wave radiation [W m^-2^], α is the albedo of the canopy, set to 0.16 (Jones, 2014). The e_air_ is the ambient water vapor pressure [kPa] and Tc the temperature of the canopy [K]. The VPD [kPa] was determined by calculating the difference of the saturation vapor pressure e_abs_ [kPa] and the actual vapor pressure e_air_ [kPa]. The actual vapor pressure was calculated using the measured relative humidity ϕ [%] and air pressure p_air_ [MPa].

$\mathbf{VPD=}\frac{\frac{\mathbf{e}_{\mathbf{air}}}{\mathbf{e}_{\mathbf{abs}}}}{\mathbf{p}_{\mathbf{air}}}$ (7)

with

$\mathbf{e}_{\mathbf{air}}\mathbf{=}\mathbf{e}^{\mathbf{(}\frac{\mathbf{52.57633-6790.4985}}{\mathbf{T}_{\mathbf{air}}}\mathbf{-5.02808}\ln\mathbf{T}_{\mathbf{air}}\mathbf{)}}$ (8)

$\mathbf{e}_{\mathbf{abs}}\mathbf{=}\mathbf{e}_{\mathbf{air}}\frac{\boldsymbol{\varphi}}{\mathbf{100}}$ (9)
